# Multiple sevoflurane exposures in early development lead to long-term vascular abnormalities in the hippocampus

**DOI:** 10.1007/s10565-026-10206-y

**Published:** 2026-05-18

**Authors:** Yu Matsumoto, Kazue Hashimoto-Torii, Masaaki Torii

**Affiliations:** 1 Center for Neuroscience Research, Children’s Research Institute, Children’s National Hospital, 111 Michigan Avenue, N.W., Washington, DC 20010-2970 USA; 2 Department of Anesthesiology & Intensive Care Medicine Graduate School of Medicine, School of Medicine, The University of Osaka, 2-2, Yamadaoka, Suita-City, Osaka 565-0871 Japan; 3 Department of Pediatrics, Pharmacology & Physiology, School of Medicine and Health Sciences, The George Washington University, Washington, DC 20052 USA

**Keywords:** Anesthesia, Hippocampus, Vasculature, Cell death, Inflammation, Anesthesia-induced neurotoxicity

## Abstract

Children who undergo multiple exposures to anesthesia for surgeries and procedures are at increased risk of developing neurocognitive impairments. Growing evidence suggests that anesthesia-induced neurotoxicity and acute damage to neural cells underlie these impairments. However, the mechanisms underlying the long-term adverse effects of early anesthetic exposure remain largely unknown. Here, we show that repeated early-life exposures to sevoflurane, a commonly used anesthetic in clinical practice, lead to long-term impairments in hippocampal vascular architecture, characterized by reduced vascular area and branching complexity, associated with alterations in postsynaptic density composition. Mechanistically, sevoflurane exposure was associated with evidence of impaired endothelial cell integrity and elevated circulating levels of the pro-inflammatory cytokine TNF-α. In vitro, physiologically relevant concentrations of TNF-α potentiated sevoflurane-induced endothelial cell injury, indicating that inflammatory signaling may exacerbate endothelial vulnerability. Together, these findings identify microvascular regression as a long-term consequence of early anesthetic exposure and suggest that preservation of endothelial integrity may represent a therapeutic strategy to mitigate or prevent anesthesia-associated neurodevelopmental risk.

**Graphical Abstract:**

• Repeated early-life exposure to sevoflurane elevates plasma TNF-α levels.

• Sevoflurane exposure reduces CD31 expression in hippocampal microvasculature.

• The combination of elevated TNF-α and endothelial vulnerability promotes vascular regression.

• Vascular regression is associated with reduced postsynaptic density.

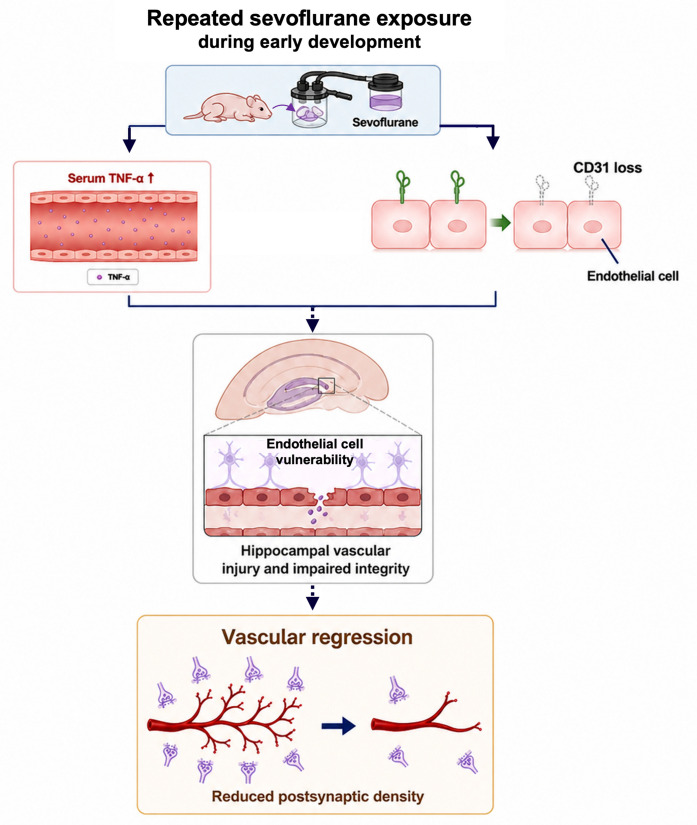

**Supplementary Information:**

The online version contains supplementary material available at 10.1007/s10565-026-10206-y.

## Introduction

Over the past few decades, animal studies have documented neurotoxic and neurodegenerative effects of anesthetics in the developing brain. This concern has prompted many retrospective studies in human infants and young children, noting a correlation between exposure to general anesthesia in infancy and subsequent neurobehavioral problems in childhood (Andropoulos 2018). This correlation is particularly evident for prolonged or repeated exposures. About 6 million children, including 1.5 million newborns and infants, receive general anesthesia each year in the United States (DeFrances et al. 2007). The possibility of anesthetic-induced neurotoxicity, therefore, is a matter of great concern and requires urgent clarification. Recent studies using animal models have reported the impacts of repeated anesthesia during early development on a broad range of neurocognitive functions, and the complex nature of anesthetic-induced neurotoxicity in the developing brain, involving various mechanisms including apoptosis, pyroptosis, and ferroptosis of neuronal and glial cells, neuroinflammation, and disrupted synaptic organization and function (Borzage and Peterson 2025). However, the mechanisms by which early exposure to anesthesia causes neurotoxicity and long-term adversity in brain function remain largely unknown.

Accumulating evidence from epidemiological and postmortem studies suggests a close relationship between small vessel disease, aberrant neurovascular regulation, and cognitive impairment. For example, patients with Alzheimer’s disease exhibit clear cerebrovascular pathology and white matter injury due to microvascular infarction (Miklossy 2003), and there is growing interest in and appreciation of pathological changes in brain microvasculature that occur before the appearance of key signatures of Alzheimer’s disease, including Aβ and tau accumulation (Wang et al. 2016). Unlike the peripheral microvasculature, the brain microvasculature not only maintains blood supply, but also facilitates interactions between neurons and glial cells (Kugler et al. 2021). Detection of changes in the brain microvasculature, therefore, is expected to facilitate early detection and intervention for progressing cognitive decline (Kisler et al. 2017).

Anesthetic agents affect cerebral microvasculature. Sevoflurane, a widely used anesthetic, has been shown to induce an influx of plasma components into the brain tissue in elderly mice 24 h after anesthesia (Acharya et al. 2015), suggesting its effect to cause an acute decline in the functional integrity of the blood–brain barrier (BBB). These mice exhibit structural changes known as luminal surface flattening of brain microvasculature and extravasated plasma components that show selective affinity for the surfaces of pyramidal neurons (Acharya et al. 2015). However, its long-term effects on cerebral microvasculature and brain function remain unknown. Furthermore, few studies have examined the effects of anesthetic administration during infancy on brain blood vessels.

In this study, we show that multiple exposures to sevoflurane during early development lead to long-lasting alterations in hippocampal vascular structure, including reduced vascular area and branch number, along with changes in postsynaptic molecular properties. Our findings support a model in which these vascular alterations involve vascular regression associated with impaired endothelial cell integrity, potentially due to the combined direct effects of sevoflurane on endothelial cells and indirect effects mediated by elevated plasma TNF-α.

## Materials and methods

### Animals

C57BL/6 J mice (The Jackson Laboratory) were maintained on a 12-h light–dark cycle (lights on 06:00–18:00) at a constant temperature (22 ± 1 °C). All animals were handled according to protocols approved by the Institutional Animal Care and Use Committee of Children’s National Hospital.

### Anesthesia

Mice from the same litter were randomly assigned to anesthesia exposure or non-exposure groups. For the exposure group, mice were anesthetized in an induction chamber with 3% sevoflurane and 40% oxygen (balanced with nitrogen) for 2 h daily over 3 consecutive days (P6-P8) using the VetFlo™ Vaporizer Single Channel Anesthesia System (Kent Scientific). For the non-exposure group, mice were similarly exposed to 40% oxygen at the identical flow rate and with the same duration of separation from their mothers. To minimize peri-anesthetic physiological instability, gas delivery was continuously maintained at a constant flow rate to ensure consistent oxygen availability. Core body temperature was actively maintained at 37 ± 0.5 °C using a warming pad placed under the chamber. During exposure, animals were continuously observed for respiratory pattern and rate, and skin color was visually monitored to assess adequate oxygenation. No animals exhibited signs of cyanosis, labored breathing, or peri-anesthetic mortality. This exposure paradigm (3% sevoflurane and 40% oxygen) has previously been reported not to significantly alter arterial blood gas parameters in neonatal mice (Sun et al. 2021), supporting maintenance of normoxic conditions under these settings. Anesthesia timing (P6-P8) was selected based on previous studies demonstrating that the mouse brain growth spurt peaks around P7, corresponding developmentally to the perinatal period in humans (Semple et al. 2013).

### Immunohistochemistry

Mice were perfused with 4% paraformaldehyde (PFA) in phosphate buffered saline (PBS) following a standard protocol. The brains were collected and post-fixed in 4% PFA at 4 °C overnight. Sagittal sections (60 µm thick) were made using a vibratome (VT1000S, Leica). Free-floating sections were treated with hydrogen peroxide in methanol (1:4) solution at −20 °C for 20 min to inactivate endogenous peroxidase activity. After washing with permeabilizing solution [PBS containing 0.05% bovine serum albumin (BSA) and 0.25% Triton X-100] three times, sections were incubated with blocking buffer (1% BSA, 0.5% Triton X-100, 2.5% normal donkey serum in PBS) for 30 min at room temperature (RT). Sections were then incubated with the primary antibodies: rat anti-CD31 (1:250; 553370, BD Biosciences), goat anti-type IV collagen (1:200; 1340–01, SouthernBiotech), rabbit anti-cleaved caspase-3 (1:400; 9661, Cell Signaling), rabbit anti-adrenomedullin (1:200; PA5-36524, Invitrogen), or rabbit anti-PSD-95 (1:300; ab18258, Abcam). After washing three times, sections were incubated with the secondary antibodies: donkey anti-rat Alexa Fluor 647 (1:600, Invitrogen), donkey anti-goat Alexa Fluor 488, (1:600, Invitrogen), horseradish peroxidase (HRP)-conjugated anti-rabbit IgG (1:600, Jackson ImmunoResearch), or HRP-conjugated anti-mouse IgG (1:600, Jackson ImmunoResearch) for 3 h at RT. TSA kit (PerkinElmer) was used for signal amplification and visualization. DAPI (4’,6-diamidino-2-phenylindole, dihydrochloride) (1:1000, Sigma-Aldrich) was used for nuclear counterstaining. Labeled sections were imaged using an Olympus confocal microscope with a digital camera.

### Quantification of plasma cytokines

Two hours after the end of the last anesthesia, blood was collected from the left ventricle of each mouse using a 25-gauge needle into a tube coated with ethylenediaminetetraacetic acid (EDTA), followed by centrifugation at 1000 × g for 10 min. Plasma was collected, aliquoted, and stored at −20 °C until use. Interleukin (IL)−6, IL-1β, and Tumor Necrosis Factor Alpha (TNF-α) levels in the plasma were quantified using a multiplex magnetic bead-based immunoassay (MYCYTOMAG-70 k, Millipore Sigma) following the manufacturer’s instructions. The specific bead fluorescence was measured using a MAGPIX Luminex instrument (Luminex). Sample concentrations were calculated using Belysa curve fitting software (Millipore Sigma).

### BMEC culture

Primary C57BL/6 mouse brain microvascular endothelial cells (BMECs) (C57-6023, Cell Biologics) were seeded in 6-well plates coated with Gelatin-Based Coating Solution (6950, Cell Biologics) and were grown to 80–90% confluency in Complete Mouse Endothelial Cell Medium with supplement kit (M1168, Cell Biologics) at 37 °C in a 5% CO_2_ incubator. All experiments were performed at passage 5. Sevoflurane and TNF-α treatments were commenced on the third day post-subculture, upon the cells reaching a confluency of approximately 70–80%.

### Treatment of cultured cells with sevoflurane

BMECs in 6-well tissue culture plates were placed in a modular incubator chamber (MIC-101, Billups-Rothenberg) equipped with an inlet and outlet, through which a gas mixture was continuously delivered. An in-line anesthetic vaporizer supplied with a gas mixture containing 21% oxygen and 5% carbon dioxide at a rate of 3 L/min was used to deliver sevoflurane for at least 10 min until 3% sevoflurane concentration was achieved. Once sealed, the chamber was placed in a 37 °C incubator for 2 h. Control cells were exposed to the air/5% carbon dioxide mixture only. The 2-h exposure procedure was repeated daily for 3 days. The culture medium was changed right before and after each treatment.

### Cell viability assay

The effects of sevoflurane and TNF-α on the viability of BMECs were tested using the MTS kit (CellTiter 96 Aqueous One Solution Cell Proliferation Assay, Promega). In brief, cells were seeded into 96-well flat-bottomed tissue culture plates (2 × 10^4^ cells/well in 100 µL) in triplicate. Cells were treated with sevoflurane and/or TNF-α for 3 consecutive days as described above. TNF-α was added in the culture medium at the concentration of 0, 10, or 20 pg/ml for 2 h, beginning concurrently with sevoflurane exposure. Following treatment, 20 µL of 3-(4,5-dimethylthiazol-2-yl)−5-(3-carboxymethoxyphenyl)−2-(4-sulfophenyl)−2H-tetrazolium (MTS) reagent (ab197010, Abcam) was added to each well and the absorbance was measured at 490 nm in a microplate reader (Synergy H4 Hybrid Reader, BioTek) 3 h later.

### Immunocytochemistry

To assess CD31 expression levels, BMECs were fixed with 4% PFA for 10 min at RT, washed three times in PBS, and blocked with 3% BSA in PBS for 1 h at RT. Cells were then incubated with rat anti-CD31 antibody (1:250, 553,370, BD Biosciences) overnight at 4 °C. After washing in PBS, cells were incubated with donkey anti-rat Alexa Fluor Plus 647 (1:600; A48272, Invitrogen) for 1 h at RT in the dark, followed by DAPI staining. Images were acquired using an Olympus confocal microscope with a digital camera.

### Western blot analysis

Proteins were extracted from primary mouse BMECs after conditioning with sevoflurane and/or TNF-α as described above. Thirty micrograms of total protein were electrophoresed on a NuPAGE 4–12% Bis–Tris gel (Invitrogen) and blotted onto polyvinylidene difluoride membranes (Invitrogen). The membranes were blocked with 5% BSA for 30 min at RT on a rotary shaker and were incubated for 1 h at RT with rabbit monoclonal anti-CD31 antibody (1:2000; ab222783, Abcam) and mouse monoclonal anti-actin antibody (1:1000; MAB1501, Chemicon), with actin used as a loading control, and exposed to HRP-conjugated secondary antibodies. The reactive protein bands were visualized by chemiluminescence on an Amersham Imager 680 (GE) using SuperSignal West Pico Plus Chemiluminescent Substrate (Thermo Fisher Scientific). Band intensities were quantified using FIJI/ImageJ software and normalized to actin.

### Image analysis

All image analyses were performed using ImageJ after the images were binarized. Images were binarized using a threshold defined based on background signal intensity. Background levels were determined from regions lacking specific staining, and the threshold was set accordingly and applied uniformly across all images within each experiment. The vascular area was calculated as the average percentage of the area covered by Collagen IV labeling in the total area of each image (635 μm × 635 μm) in the CA1 region of the hippocampus. The total microvascular length and branch number were quantified using the ImageJ script previously described (Rust et al. 2020). Vessel segment length and vascular branching were assessed by skeletonizing the binary image. This approach allows all pixels in a skeleton image to be tagged, all branches to be counted, and their length to be measured. The pericyte coverage of blood vessels was calculated as the average percentage of the area fraction of CD13 labeling relative to Collagen IV labeling. The numbers of Collagen IV^+^/CD31^−^ blood vessels and endothelial tip cells in the total area of each image (635 μm × 635 μm) in the hippocampal CA1 were counted manually. The immunoreactive areas of IgG, cleaved caspase-3, and PSD-95 were quantified as the percentages of the labeled areas in the total area of each image (1024 µm × 1024 µm) in the hippocampal CA1. For the analysis of intercellular gaps between endothelial cells cultured in vitro (Fig. 7), the percentages of the area that were not covered by endothelial cells in the total area of each image (1272 μm × 1272 μm) were quantified. The researcher was blinded to treatment for these analyses.

### Statistical analysis

GraphPad Prism (version 10.1.0) was used to generate all graphs and perform statistical analyses. Data normality was assessed by the Shapiro–Wilk test. As we found that all data in our study followed a normal distribution, parametric tests were used. A two-tailed Welch’s t-test was used for two-group comparisons of data. For comparisons involving two independent variables, two-way analysis of variance (ANOVA) followed by a post-hoc Šidák test was performed. Pearson’s correlation coefficients were calculated to assess associations between variables. Outliers were assessed using the ROUT (Robust regression and Outlier removal) method implemented in GraphPad Prism. We found no outliers in any of the data. The number of samples (n) for each analysis is provided in the figure legends. For histological image-based analyses, measurements obtained from two sections from the same animal were averaged to generate a single representative value per animal. Data are represented as the mean ± SEM. P values < 0.05 were considered statistically significant. Nonsignificant results should be interpreted with caution, particularly in analyses with relatively small sample sizes, where limited statistical power (Type II error) cannot be excluded.

## Results

### Reduced microvascular density and complexity in the mouse hippocampus following multiple neonatal exposures to sevoflurane

**Fig. 1:**
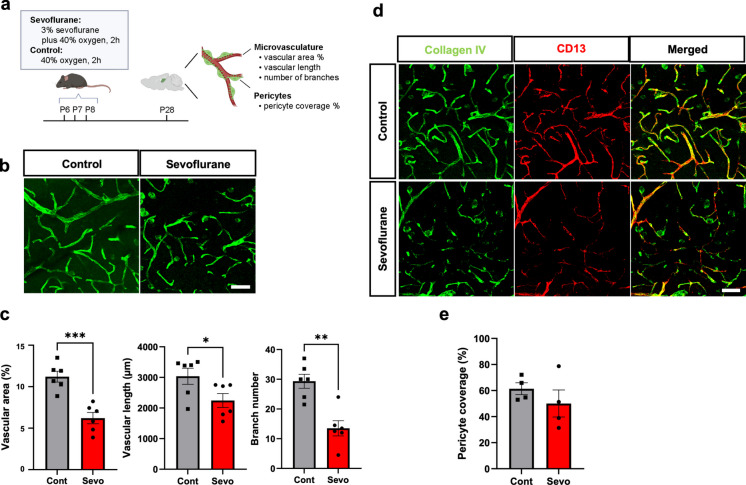
Long-term microvascular changes in the hippocampus following multiple exposures to sevoflurane. (**a**) Experimental design. (**b**) Representative images of hippocampal (CA1) microvasculature visualized by immunohistochemistry for Collagen IV, a marker of the vascular basement membrane, in control (non-exposed) and sevoflurane-exposed mice at PAD28. Scale bar: 50 µm. (**c**) Quantitative assessment of microvascular parameters (vascular area, total vascular length, and branch number) reveals significant differences between control and sevoflurane-exposed groups; ***P = 0.0004, *P = 0.0454, and **P = 0.0011, respectively (*n* = 6 animals per group; two-tailed Welch’s t-test). (**d**) Representative images of hippocampal (CA1) microvasculature labeled for Collagen IV (green) and CD13 (red; pericyte marker) in sevoflurane-exposed and non-exposed mice at PAD28. Scale bar: 100 µm. (**e**) Quantitative assessment of pericyte coverage of the microvasculature; P = 0.373 (*n* = 4 animals per group; two-tailed Welch’s t-test)

We first examined whether multiple exposures to sevoflurane during early development cause long-term structural changes in brain microvasculature. Since the peak of the brain growth spurt is around birth in humans and around P7 in mice (Semple et al. 2013), we anesthetized mice with 3% sevoflurane in 40% oxygen (or 40% oxygen as the control) for 2 h daily for 3 days at P6, P7, and P8, and examined the brain at post anesthesia day (PAD) 28 (Fig. 1a), which corresponds to early adolescence (in mice), a developmental stage at which many clinical studies have reported neurotoxicity of anesthesia. Brain vasculature was visualized by immunohistochemistry (IHC) for Collagen IV, a marker for the basement membrane of blood vessels, and the structure was assessed in the hippocampal CA1 region, where the most prominent changes were observed.

In sevoflurane-exposed animals, the vascular area, total vascular length, and branch numbers were reduced compared with controls (Fig. 1b, c). The percentage of pericyte coverage, which is an important parameter for evaluating physiological function and maturity of blood vessels (Gautam and Yao 2018; Zlokovic 2010), was not significantly different between sevoflurane-exposed and control groups (Fig. 1d, e). These results indicate that the overall vascular density and branch complexity are reduced in the hippocampus following neonatal sevoflurane exposure and remain lower several weeks after anesthesia, while pericyte coverage of the remaining vessels appears to be preserved.

### Reduction of postsynaptic density associated with the reduced vascular density

**Fig. 2:**
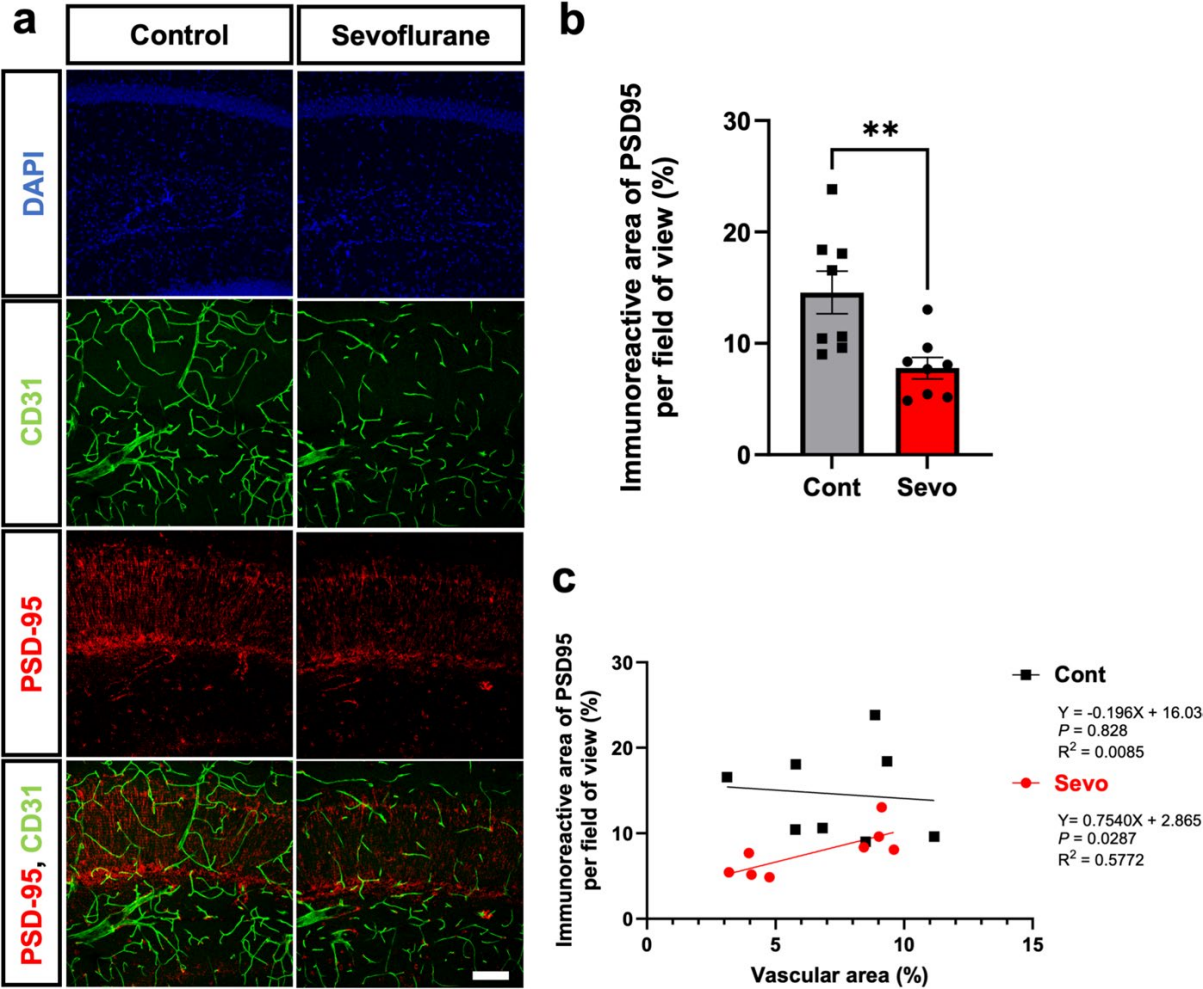
Reduction of PSD-95 correlated with reduced vascular area in the hippocampus of sevoflurane-exposed mice. (**a**) Representative images showing PSD-95^+^ postsynaptic densities (red) and CD31^+^ vascular endothelial cells (green) in the CA1 in control and sevoflurane-exposed mice at PAD28. Scale bar: 100 µm. (**b**) Quantification reveals a decrease in the PSD-95 immunoreactive area in the CA1 of sevoflurane-exposed mice compared with controls; **P = 0.0096 (*n* = 8 animals per group; two-tailed Welch’s t-test). (**c**) Pearson’s correlation analysis (exploratory due to sample size) reveals a positive correlation between PSD-95 immunoreactive area and vascular area in the CA1 region of sevoflurane-exposed mice (Pearson’s coefficient of determination R^2^ = 0.5772, P = 0.0287), but not in control mice (R^2^ = 0.0085, P = 0.828). Slopes were compared using linear regression with an interaction term, revealing a significant difference between groups; P = 0.049. Each data point represents one animal

Vascular abnormalities such as endothelial dysfunction and reduced cerebral blood flow exacerbate neuronal and synaptic injury through neurovascular uncoupling (Tarawneh 2023). Lower neurovascular coupling in the hippocampus than in the neocortex has also been suggested to contribute to the higher vulnerability of the hippocampus to decreased energy supply in neurological diseases (Shaw et al. 2021). To assess potential synaptic abnormalities, we examined the expression of postsynaptic density protein 95 (PSD-95), a major scaffolding protein in the mature postsynaptic density and a potent regulator of synaptic strength (Ehrlich et al. 2007). IHC on PAD28 revealed a significant decrease of PSD-95 in the CA1 in sevoflurane-exposed mice compared with control mice (Fig. 2a, b). A positive correlation was found between vascular density and the density of PSD-95 immunoreactivity in the CA1 of sevoflurane-exposed mice (Fig. 2c), although these analyses are exploratory in nature due to the limited sample size. These results suggest that reduced microvascular density following multiple exposures to sevoflurane may contribute to impaired synaptic function in the hippocampus.

### Normal angiogenesis but increased vascular regression after multiple exposures to sevoflurane

**Fig. 3:**
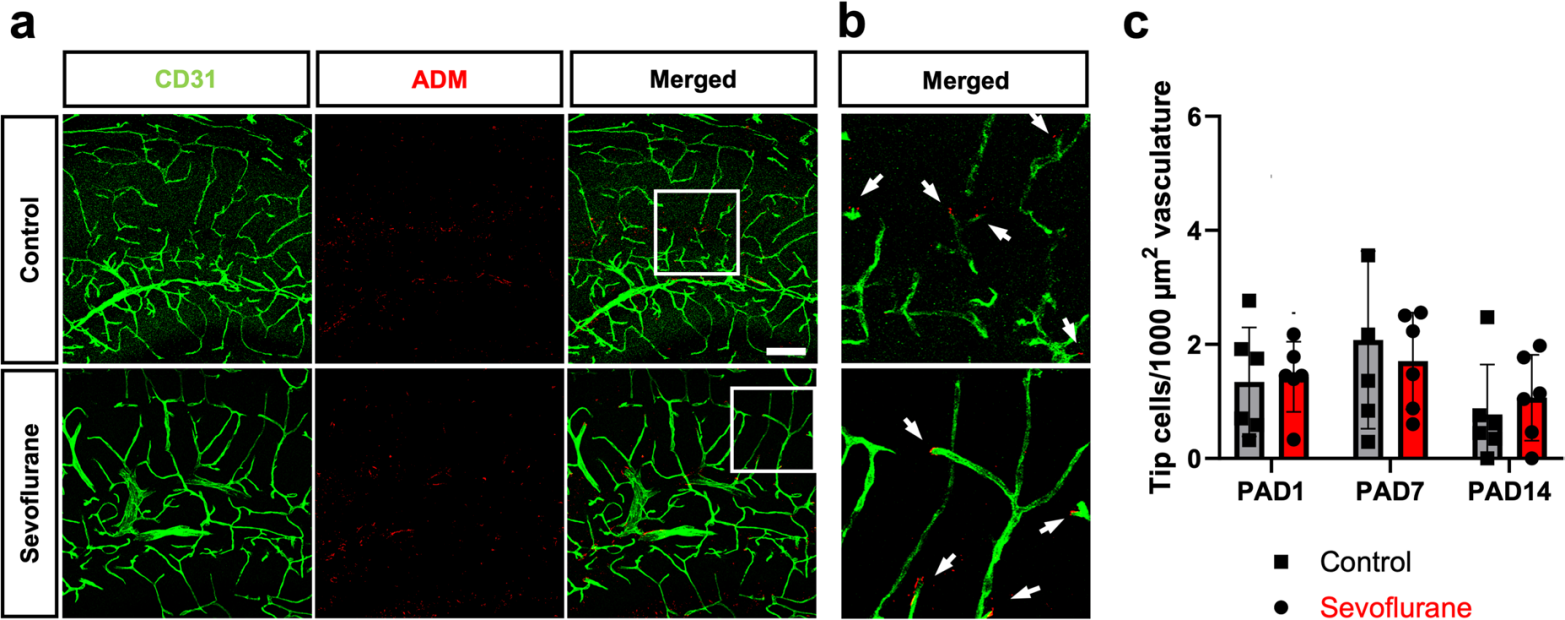
Multiple exposures to sevoflurane do not show significant effects on angiogenesis in the CA1 region of the neonatal hippocampus. (**a**) Representative images of hippocampal (CA1) microvasculature and endothelial tip cells immunofluorescently labeled for CD31 (green) and ADM (red), respectively, in control and sevoflurane-exposed mice at PAD1. Scale bar: 100 µm. (**b**) Higher-magnification views of the boxed areas in (a). The arrows point to ADM^+^ endothelial tip cells at the vascular front. (**c**) Quantitative analysis of tip cell numbers at PAD1, 7, and 14, showing no statistically significant differences detected between control and sevoflurane-exposed mice; P = 0.8518, 0.6246, and 0.5484, respectively (*n* = 6 animals per group at each time point; two-tailed Welch’s t-test)

The vascular network of the brain parenchyma is established via sprouting angiogenesis, which is driven by endothelial tip cells, a subset of endothelial cells found at the leading edge of the vascular sprout (Fantin et al. 2015, 2013). Sprouting angiogenesis occurs not only during embryonic development but also in the early postnatal period (Wälchli et al. 2013). To investigate whether a decrease in sprouting angiogenesis is involved in the reduced vascular density and complexity in the hippocampus after anesthesia, we compared the number of endothelial tip cells at the vascular front between anesthesia-exposed and control animals by IHC for the tip cell marker adrenomedullin (ADM) and the endothelial cell marker CD31 at PAD1, 7, and 14. Tip cell counts were similar between control and sevoflurane-exposed groups, and we did not detect a significant difference under these conditions (Fig. 3a-c).

**Fig. 4:**
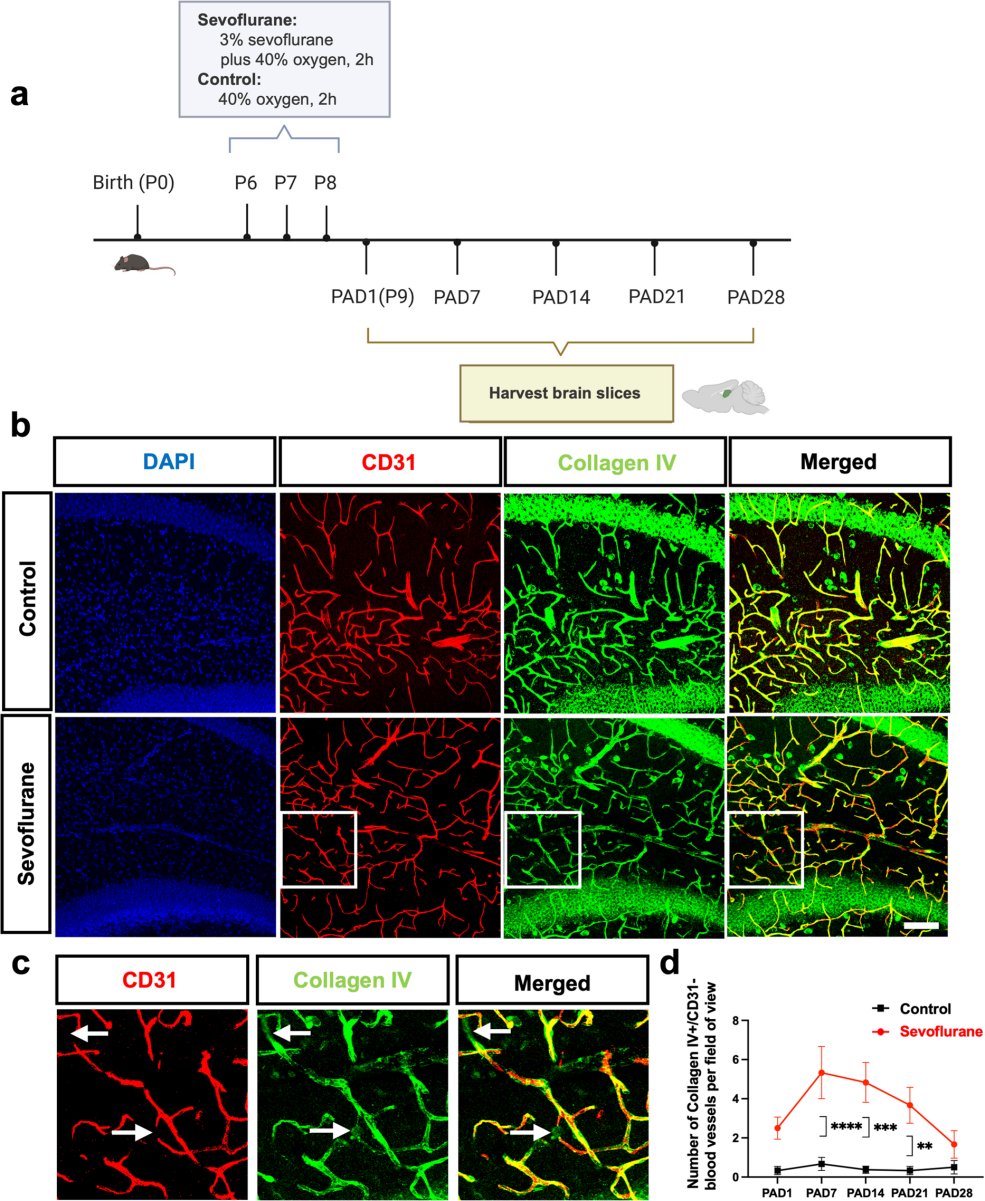
Loss of CD31 immunoreactivity in portions of the hippocampal microvasculature in sevoflurane-exposed mice. (**a**) Experimental timeline. (**b**) Representative images showing the expression of CD31 (red) and Collagen IV (green) in the CA1 region of the hippocampus at PAD7. Scale bar: 100 µm. (**c**) Higher-magnification views of the boxed area in (**b**). The arrows point to the regions of putative blood vessel regression, which exhibits vascular scaffolding that lack CD31 immunoreactivity. (**d**) The number of CD31^−^/Collagen IV^+^ blood vessels in sevoflurane-exposed mice is higher than that in controls from PAD1, showing statistically significant differences from PAD7 through PAD21 and returning to control levels by PAD28; no significant interaction between the effects of sevoflurane exposure and PAD [F(4,50) = 2.32, *P* = 0.069], but significant main effects of sevoflurane exposure [F(1,50) = 51.83, P < 0.0001] and PAD [F(4,50) = 2.643, P = 0.044] were found (n = 6 animals per PAD per group; two-way ANOVA). ****P < 0.0001 (PAD7), ***P = 0.0002 (PAD14), and **P = 0.0067 (PAD21) (Šidák test)

Another major mechanism that potentially contributes to the reduced vascular density and complexity is vascular regression (Bickel et al. 2023). We, therefore, assessed vascular regression in the CA1 region by quantifying Collagen IV^+^ blood vessels lacking CD31^+^ endothelial labeling, which has been used as an indicator of vessel regression (Lee et al. 2018) (Fig. 4a). The number of Collagen IV^+^ microvessels lacking CD31 labeling in sevoflurane-exposed mice was greater than that in controls even at PAD1, with statistically significant differences peaking from PAD7 through PAD21, and returning to the control level by PAD28 (Fig. 4b-d). These results suggest that pathological regression of microvessels in the neonatal hippocampus starts immediately following multiple exposures to sevoflurane, persists for approximately three weeks, and may contribute to a long-term decrease in vascular density.

### Involvement of endothelial cell apoptosis in blood vessel regression following multiple exposures to sevoflurane

**Fig. 5:**
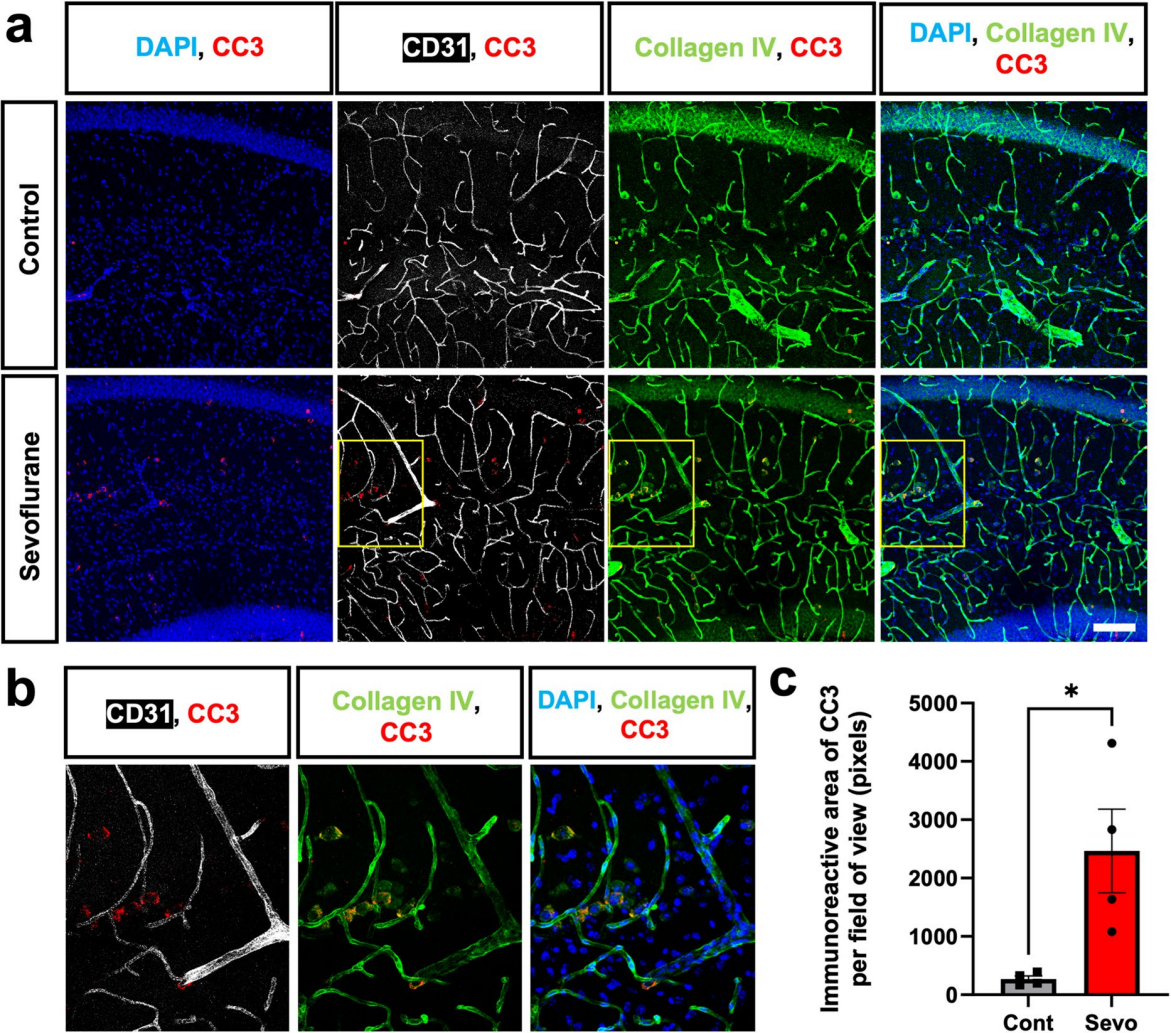
Increased apoptosis in the hippocampal microvasculature in sevoflurane-exposed mice. (**a**) Representative images of quadruple immunofluorescence labeling for CD31 (gray), cleaved caspase-3 (CC3, red), Collagen IV (green), and DAPI (blue) in the CA1 region in control and sevoflurane-exposed mice at PAD7. Scale bar: 100 µm. (**b**) Higher-magnification views of the boxed areas in (**a**). Localization of CC3 in Collagen IV^+^/CD31^−^ microvascular regions is observed, often accompanied by Collagen IV^+^/CD31^−^ cells showing perinuclear CC3 labeling. (**c**) CC3 immunoreactive area in the CA1 region appears significantly increased in sevoflurane-exposed mice than in controls; *P = 0.0223 (n = 4 animals per group; two-tailed Welch’s t-test)

Apoptotic cell death has been identified as a major mechanism underlying endothelial cell loss during blood vessel regression (Korn and Augustin 2015). As we observed microvessels that exhibited hallmarks of regression in the hippocampus of sevoflurane-exposed animals (Fig. 4), we examined apoptotic cell death by detecting the activation of caspase-3, a key mediator of apoptosis. Cleaved (i.e., activated) caspase-3 immunoreactivity was significantly increased in the CA1 region in sevoflurane-exposed mice compared with controls on PAD7 (Fig. 5a, c), particularly in cells adjacent to constricted vessels that were likely undergoing regression (Korn and Augustin 2015)(Fig. 5b). These results suggest that apoptosis in putative endothelial cells may contribute to vascular regression; however, because apoptotic cell identity was not definitively established, contributions from non-endothelial cell apoptosis cannot be excluded, nor can the involvement of other mechanisms such as endothelial cell migration (Selvam et al. 2018).

### Blood–brain barrier disruption during blood vessel regression

**Fig. 6:**
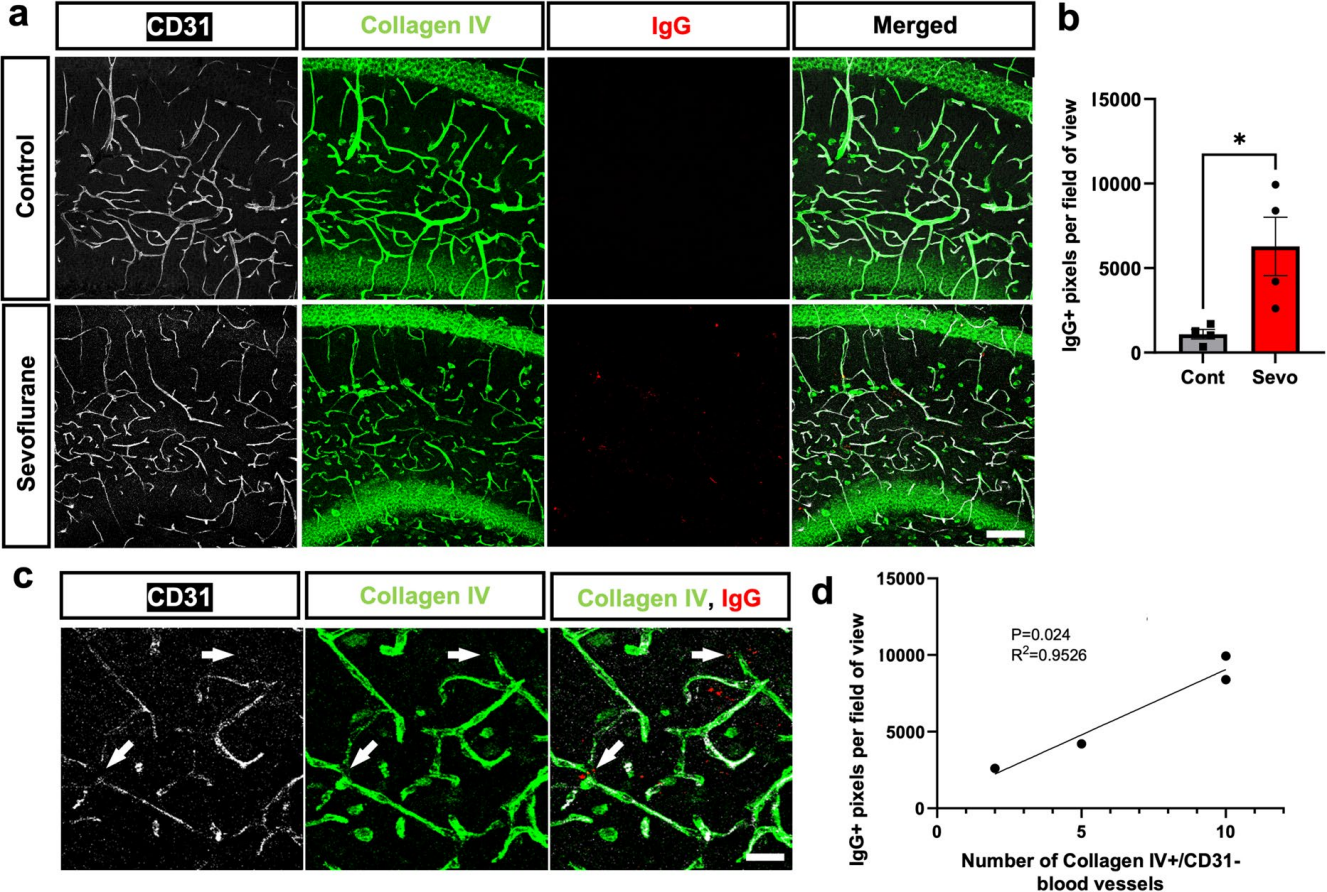
Blood brain barrier alterations in the hippocampus of sevoflurane-exposed mice. (**a**) Representative images of the hippocampal CA1 region immunofluorescently labeled for CD31 (gray), Collagen IV (green), and IgG (red) in control and sevoflurane-exposed mice at PAD7. Scale bar: 100 µm. (**b**) Quantitative analysis shows an increase in IgG deposits following sevoflurane exposure; *P = 0.0455 (n = 4 animals per group; two-tailed Welch’s t-test). (**c**) Higher-magnification views in the CA1, showing extravascular IgG deposits (arrows) around Collagen IV^+^/CD31^−^ blood vessels. Scale bar: 25 µm. (**d**) Pearson’s correlation analysis (exploratory due to sample size) reveals a positive correlation between the number of Collagen IV^+^/CD31^−^ blood vessels and IgG deposit area in the CA1 region; R^2^ = 0.95326, *P* = 0.024 (n = 4 animals per group)

CD31, also known as PECAM-1 (platelet endothelial adhesion molecule), is a member of the immunoglobulin gene superfamily expressed at high density at the lateral borders of endothelial cells (Muller 2003), and its deficiency is associated with excessive vascular leakage (Cheung et al. 2020). We, therefore, investigated the possibility that, in addition to vascular regression and putative endothelial cell loss, sevoflurane exposure affects the functional integrity of the BBB. Leakage of plasma components from blood vessels was assessed by IHC for IgG, which is normally absent from the brain interstitial space when the BBB is functional (Acharya et al. 2015). We detected a significantly higher incidence of extravascular IgG leakage in sevoflurane-exposed animals compared with controls on PAD7 (Fig. 6a, b). The leakage was often found around CD31^−^ microvessels (Fig. 6c). Pearson’s correlation analysis revealed a positive correlation between the number of Collagen IV^+^/CD31^−^ blood vessels and the area of IgG deposition in the CA1 region (Fig. 6d) although these analyses are exploratory in nature due to the limited sample size. These results suggest that the BBB integrity may be compromised in association with reduced CD31 expression and/or endothelial alterations during vascular regression.

### Direct effects of sevoflurane on endothelial CD31 expression

**Fig. 7:**
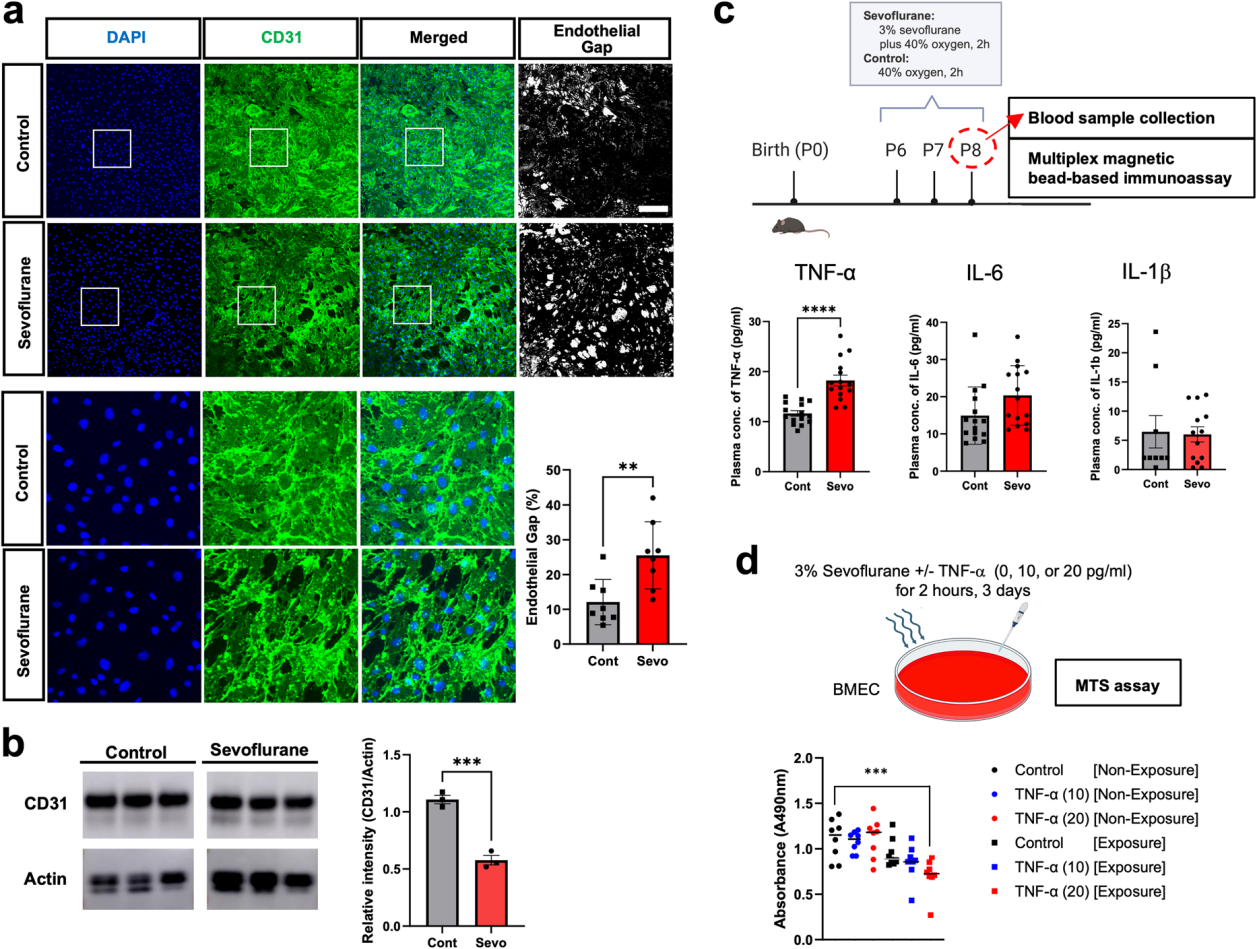
Direct and indirect effects of sevoflurane on endothelial cells associated with endothelial cell death. (**a**) Immunocytochemical analysis of the impact of sevoflurane exposure on endothelial cell adhesion. Endothelial cell intercellular junctions are visualized by enriched expression of CD31 (green). The bottom six panels show higher-magnification views of the boxed areas in the top panels. The percent area of endothelial gaps (transient openings between endothelial cells) is significantly larger in sevoflurane-exposed cultures than in non-exposed (control) cultures; **P = 0.0066 (*n* = 8 independent culture preparations per group; two-tailed Welch’s t-test). Scale bar: 300 µm. (**b**) Western blot analysis of CD31 protein levels in BMECs in control and sevoflurane-exposed cultures. CD31 levels (relative intensity), normalized to actin, are significantly lower in sevoflurane-exposed BMECs than in non-exposed BMECs; ***P = 0.0007 (*n* = 3 independent culture preparations per group; two-tailed Welch’s t-test). (**c**) Multiplex magnetic-bead based immunoassay assessing the effect of neonatal sevoflurane exposure on plasma levels of proinflammatory cytokines (TNF-α, IL-6, and IL-1β). Blood samples were collected at P8 (2 h after the last exposure to sevoflurane or control conditions). Plasma TNF-α levels are significantly elevated in sevoflurane-exposed mice compared with non-exposed mice, while IL-6 and IL-1β levels show no significant differences between the two groups; ****P < 0.0001, 0.0682, and 0.8856, respectively (*n* = 15 animals per group; two-tailed Welch’s t-test). (**d**) MTS assay examining the combinatorial effects of sevoflurane and TNF-α on BMEC viability in vitro. Cell viability, assessed by absorbance in the MTS assay, shows a significant decrease only in cultures exposed to both sevoflurane exposure and 20 pg/ml TNF-α. No significant interaction between the effects of sevoflurane exposure and TNF-α was found by two-way ANOVA [F(2,42) = 2.16, *P* = 0.13]. A significant main effect of sevoflurane exposure [F(1,42) = 23.49, *P* < 0.0001] but not of TNF-α [F(2,42) = 1.572, *P* = 0.22] was observed. ***P = 0.0006 by Šidák test (*n* = 8 independent experiments, each performed in triplicate, per group)

To clarify whether sevoflurane exposure acts directly on endothelial cells to decrease CD31, we examined the effects of sevoflurane exposure on CD31 expression in primary mouse brain microvascular endothelial cells (BMECs) (Fig. 7a). Cells were exposed to 3% sevoflurane or control gas in a 37 °C incubator for 2 h daily for 3 days. In control cultures, immunocytochemistry for CD31 showed a characteristic jagged pattern, with high at presumably adherent junctions where CD31 homodimers form across adjacent cell membranes (Zhao and Newman 2001) (Fig. 7a). In contrast, BMECs exposed to sevoflurane showed a significant increase in the area of gaps between adjacent cells, in addition to an overall reduction in CD31 immunoreactivity (Fig. 7a), suggesting impairment of intercellular adhesion associated with decreased CD31 levels. Decreased expression of CD31 in sevoflurane-exposed BMECs was quantitatively confirmed by Immunoblotting with whole-cell lysates (Fig. 7b).

### Induction of endothelial cell death by combined exposure to sevoflurane and elevated TNF-α

Previous studies have reported that CD31 also plays a role in protecting endothelial cells from cell death induced by proinflammatory cytokines (Cheung et al. 2015), and sevoflurane exposure elevates plasma levels of proinflammatory cytokines (Inoue et al. 2016). Since we observed CD31 downregulation in BMECs by sevoflurane exposure alone (Fig. 7a, b), we hypothesized that sevoflurane exposure-induced endothelial cell apoptosis is mediated by the combined direct effects of sevoflurane on vascular endothelial cells and indirect effects of proinflammatory cytokines, potentially elevated by multiple sevoflurane exposures in neonates.

Plasma concentrations of proinflammatory cytokines in sevoflurane-exposed and control groups of mice were measured at P8 (2 h after the last exposure to sevoflurane or control gas mixture) using a multiplex magnetic bead-based immunoassay (Fig. 7c). The sevoflurane-exposed group showed a significant increase in the concentration of Tumor Necrosis Factor alpha (TNF-α) compared with the control group, while no significant differences were detected in the plasma levels of other proinflammatory cytokines, Interleukin (IL)−6 and IL-1β between the two groups (Fig. 7c).

To determine whether sevoflurane and/or TNF-α alone or in combination, induces endothelial cell death, we then examined their effects on cell viability in vitro using the MTS assay (Fig. 7d). Based on the plasma concentration of TNF-α in sevoflurane-exposed mice (Fig. 7c), three concentrations of TNF-α (0, 10, and 20 pg/ml) were tested with and without sevoflurane exposure (2 h daily for 3 days). The viability of BMECs, measured based on the absorbance reading at 490 nm, was found to be significantly lower with the combination of sevoflurane exposure and TNF-α treatment at 20 pg/ml compared to the control (0 pg/ml TNF-α without sevoflurane exposure), while no significant effects were observed with the other combinations (Fig. 7d). These results support a model in which the combination of direct effects of sevoflurane and indirect effects associated with sevoflurane-induced elevation of TNF-α promotes endothelial cell death.

## Discussion

This study provides evidence that multiple exposures to sevoflurane during early brain development lead to long-term reductions in vascular density and complexity, as well as alterations in postsynaptic markers within the hippocampus. These adverse effects are consistent with a model involving BBB dysfunction and vascular regression, potentially linked to endothelial vulnerability and inflammatory signaling following repeated sevoflurane exposure. Together, these findings suggest a possible mechanistic pathway by which repeated exposure to general anesthesia during infancy may increase the risk of later neurodevelopmental deficits.

Since the maintenance of normal brain function relies on a stable blood supply, which is closely linked to vascular features, such as vascular area, length, and branch number (Wei et al. 2021), these features are commonly employed to assess functional abnormalities in neurodegenerative diseases and aging (Chen et al. 2021). Studies using magnetic resonance imaging (MRI) have revealed that individuals with mild cognitive impairment exhibit smaller hippocampal vascular volume compared with healthy individuals, with a positive correlation between vascular density and cognitive assessment scores (Perosa et al. 2020; Wang et al. 2006). The reduced vascular density and branch number observed in the hippocampus following repeated early-life sevoflurane exposure may be associated with altered cerebral perfusion, and thereby could contribute to subsequent neurobehavioral vulnerability (Andropoulos 2018). Since vascular endothelial cells not only provide an anatomical and physiological barrier but also serve as a highly active metabolic system that synthesizes various materials to nourish nerves and regulate vasomotor function (Wang et al. 2016), a decrease in endothelial cell number could impair brain function through disturbances in this metabolism. The reduction of PSD-95 immunoreactive area in correlation with the decreased vascular density (Fig. 2) is consistent with these possibilities. PSD-95 downregulation in the hippocampus that occurs concurrently with cognitive impairment has also been shown by immunoblotting in previous studies using similar anesthesia models in juvenile mice (Lu et al. 2017; Zhang et al. 2015). Although these associations are correlative, they are consistent with existing literature linking hippocampal vascular integrity to synaptic maintenance and function (Schaeffer and Iadecola 2021).

Our results are consistent with previous reports that the hippocampus is extremely vulnerable to various insults such as ischemia, hypoglycemia, and anoxia, and that a tightly controlled blood supply is particularly crucial in this brain region (Bartsch et al. 2015). Importantly, many studies have reported that repeated exposures to sevoflurane during early development are particularly detrimental to the hippocampus at the molecular, cellular, and functional levels (Borzage and Peterson 2025). The neurovascular unit, consisting of vasculature, neurons, and glia, plays a key role in maintaining tight control of blood supply through neurovascular coupling (Yu et al. 2020). As the hippocampus is a brain region with characteristically low neurovascular coupling (Shaw et al. 2021), a slight decrease in vascular density or function may have particularly marked impacts on this region.

The primary vascular networks of arterioles and venules in the brain are largely set during the embryonic stage, whereas most subsurface capillary beds are established during postnatal development in rodents (i.e., the second phase of angiogenesis) (Coelho-Santos et al. 2021), which corresponds to the period of sevoflurane exposure in this study. However, we did not detect significant differences in angiogenesis as evaluated by tip cell counts between anesthesia-exposed and control groups. This contrasts with previous reports that sevoflurane inhibits angiogenesis in the postnatal mouse retina by suppressing VEGF expression (Kim et al. 2018) and in human tumor cells by suppressing Ras and Rac1 signaling (Wang et al. 2020). This discrepancy may be attributed to differences in experimental paradigms, e.g., hippocampus (our study) vs retina or tumor cells, and/or in vivo (our study) vs in vitro analyses. Subtle effects in angiogenesis, however, cannot be excluded given the limited sample size of our study; future studies with larger sample sizes and additional angiogenic readouts will be needed.

On the other hand, blood vessels in the hippocampus of sevoflurane-exposed animals exhibited features consistent with vascular regression, including irregular vascular thickness and reduced CD31 immunoreactivity while retaining Collagen IV^+^ basement membrane structures (Lee et al. 2018). Because loss of CD31 immunoreactivity does not necessarily indicate endothelial cell loss, these findings may reflect endothelial phenotypic changes and/or vascular remodeling rather than complete endothelial depletion. We also observed IgG leakage into the region surrounding these regressing vessels, consistent with previous studies reporting microvascular leakage due to CD31 deficiency (Cheung et al. 2020) and the role of CD31 in endothelial cell adhesion (Lertkiatmongkol et al. 2016). Although a single exposure to sevoflurane has been reported to induce BBB disruption that triggers chronic leakage of plasma components and the homeostatic breakdown in the brain of elderly mice (Acharya et al. 2015), BBB disruption by a single exposure to sevoflurane during the neonatal period has been shown to recover within 48 h (Sun et al. 2019). Our results from multiple exposures of neonatal mice to sevoflurane showed a pattern similar to the latter, with CD31 loss (observed from PAD1 to PAD21) associated with BBB disruption peaking at PAD7 and subsequently returning to baseline levels. Similarly, despite the overall reduction in vascular density and branch complexity weeks after anesthesia, the remaining blood vessels show healthy maturation (Fig. 1), indicating significant resilience to BBB injury in the developing brain.

In light of the in vitro findings (Fig. 7), the decreased vascular density/complexity observed alongside the appearance of Collagen IV^+^/CD31^−^ vascular segments, together with increased cleaved caspase-3 immunoreactivity in these regions, suggest the involvement of apoptotic processes in putative endothelial cells during vascular regression, although formal confirmatory studies are required to definitively identify the cell type(s) undergoing apoptosis. In response to various internal or external stimuli (O'Brien and Kirby 2008), the extrinsic pathway of apoptosis is triggered by the aggregation and activation of death receptors (DRs) that belong to the tumor necrosis factor (TNF) receptor superfamily, when the extracellular concentration of the ligands reaches a particular threshold (Galluzzi et al. 2012). Indeed, we found a significant increase in the plasma level of TNF-α, a ligand for a DR, TNFR1 (Ashkenazi and Dixit 1998), in sevoflurane-exposed neonatal animals. Previous studies have reported that repeated exposures to sevoflurane cause neuroinflammation and lead to the production of proinflammatory cytokines in the brain, including TNF-α in the hippocampus (Dai et al. 2021; Shen et al. 2013). As evidence for interactions between systemic inflammation and neuroinflammation continues to accumulate (Sun et al. 2022), it will be important to determine whether the increase in plasma TNF-α in response to multiple sevoflurane exposures is initially derived from neuroinflammation or from a systemic inflammatory response. Together, these findings support a model in which TNF‑α increases endothelial vulnerability by promoting apoptotic signaling and barrier disruption during repeated sevoflurane exposure. In the developing brain, such effects may be especially consequential during periods of rapid synaptic maturation and high metabolic demand.

Our results reveal that TNF-α treatment alone or direct exposure to sevoflurane alone does not affect the viability of primary BMECs in vitro, while the combination of these two reduces their viability. As sevoflurane alone led to a decrease in CD31, which plays a role in protecting endothelial cells from cell death induced by proinflammatory cytokines including TNF-α (Cheung et al. 2015), these results support a model in which sevoflurane exposure, together with elevated inflammatory cytokines such as TNF-α, may increase endothelial vulnerability to cell death. How sevoflurane reduces CD31 in vascular endothelial cells awaits further studies, but inhibition of NMDA-dependent regulation of CD31 (Kim et al. 2022) is one possibility. A study on the postnatal development of retinal vasculature has shown decreases in retinal vasculature density and endothelial cell numbers, as well as an increase in the rate of apoptosis in retinal vessels of CD31-/- mice (Dimaio et al. 2008). Therefore, we cannot completely exclude the possibility that, in vivo, CD31 downregulation alone may be sufficient to cause a reduction in hippocampal vascular density and loss of endothelial cells. Similarly, a study has shown that TNF-α alone can lead to endothelial cell death and vascular regression in vitro albeit at a much higher concentration (Koller et al. 2020).

In conclusion, our findings indicate that multiple exposures to sevoflurane during early development are associated with vascular regression and long-lasting alterations in hippocampal microvascular structure, which may contribute to synaptic abnormalities. While this study has limitations, including relatively modest sample sizes and correlative measures in some analyses, the consistency of the findings across independent in vivo and in vitro approaches supports the interpretation that endothelial vulnerability and microvascular alterations are involved. Current approaches aimed at preventing or alleviating the neurotoxicity of neonatal exposure to general anesthetics largely focus on molecular and cellular processes in neural cells. Our findings highlight the microvasculature and endothelial cells as additional contributors to anesthesia-associated neurodevelopmental vulnerability and suggest that vascular-protective strategies warrant further investigation.

## Supplementary Information

Below is the link to the electronic supplementary material.

Supplementary file1 (DOCX 473 KB)

## Data Availability

All data is included in the manuscript and supporting information.
