# Supplementary material for: Multiple sevoflurane exposures in early development lead to long-term vascular abnormalities in the hippocampus
Source: Cell Biol Toxicol. 2026 May 18;42(1):88. doi: 10.1007/s10565-026-10206-y (PMC13350219; doi:10.1007/s10565-026-10206-y)
Supplement: Supplementary file 1 — Supplementary file1 (DOCX 473 KB) [file 10565_2026_10206_MOESM1_ESM.docx]

**Supplementary Information - Cell Biology and Toxicology**

**Multiple sevoflurane exposures in early development lead to long-term vascular abnormalities in the hippocampus**

Yu Matsumoto^1,2^, Kazue Hashimoto-Torii^1,3^, Masaaki Torii^1,3,*^

^1^Center for Neuroscience Research, Children's Research Institute, Children's National Hospital, Washington, DC 20010, USA.

^2^Department of Anesthesiology & Intensive Care Medicine Graduate School of Medicine, School of Medicine, The University of Osaka, 2-2, Yamadaoka, Suita-city, Osaka 565-0871, Japan.

^3^Department of Pediatrics, Pharmacology & Physiology, School of Medicine and Health Sciences, The George Washington University, Washington, DC, 20052, USA.

*To whom correspondence may be addressed:

Masaaki Torii

111 Michigan Avenue, N.W., M7631

Washington DC, 20010-2970

Phone: 202-476-4279

Email: mtorii@childrensnational.org

**Supplementary Figure**

**
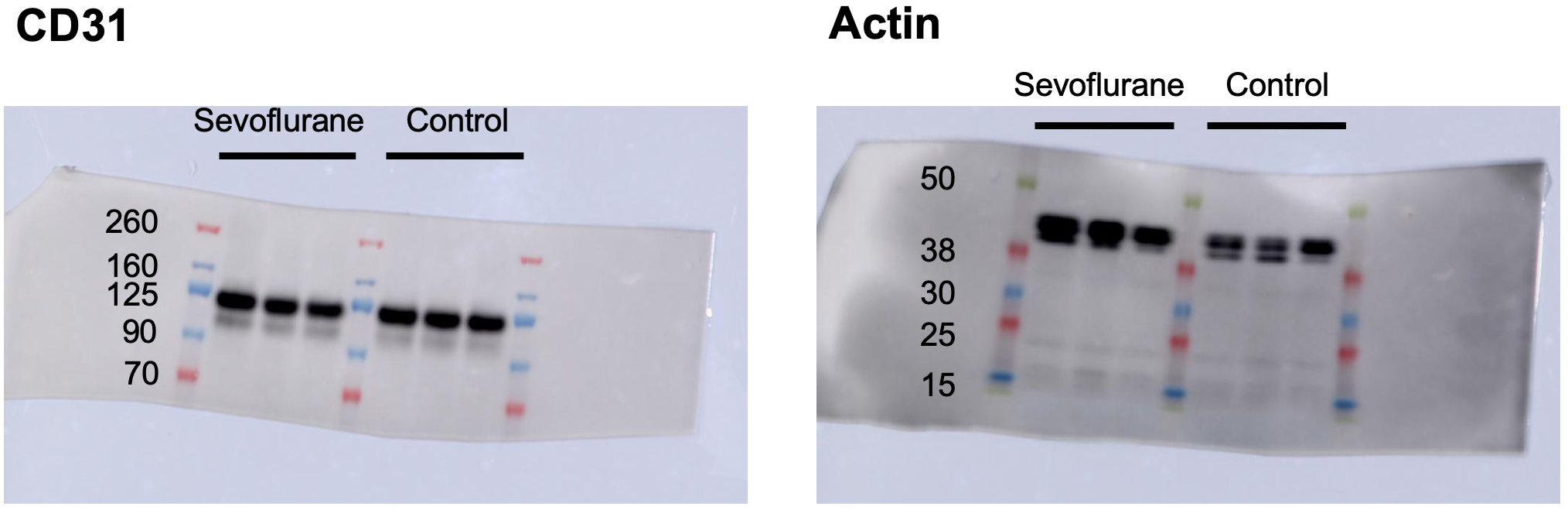
**

**Supplementary Figure 1. Full uncropped gel and blot images for Figure 7b**

Full uncropped images of Western blot analysis of CD31 and actin protein levels in BMECs in sevoflurane-exposed and non-exposed cultures.
